# 3D printed rectal swabs for assessing the gut microbiome, metabolome and inflammation

**DOI:** 10.1038/s41598-024-67457-0

**Published:** 2024-07-18

**Authors:** Robert W. Perry, Benjamin H. Mullish, James L. Alexander, Raashi Shah, Nathan P. Danckert, Jesus Miguens Blanco, Lauren Roberts, Zhigang Liu, Despoina Chrysostomou, Shiva T. Radhakrishnan, Sharmili Balarajah, Rachael Barry, Lucy C. Hicks, Horace R. T. Williams, Julian R. Marchesi

**Affiliations:** 1 Department of Metabolism, Digestion and Reproduction, Faculty of Medicine, Imperial College London, London, UK; 2 Departments of Gastroenterology and Hepatology, St Mary’s Hospital, Imperial College Healthcare NHS Trust, London, UK; 3 Department of Twin Research and Genetic Epidemiology, School of Life Course & Population Sciences, King’s College London, London, UK; 4 Department of Bioengineering, Stanford University, Stanford, CA USA; 5 Department of Gastroenterology, St Marks Hospital, London, UK

**Keywords:** Microbiology, Gastroenterology

## Abstract

Investigating the gut microbiome and metabolome frequently requires faecal samples, which can be difficult to obtain. Previous studies have shown that rectal swabs are comparable to faecal samples for analysing gut microbiota composition and key metabolites. In this study, 3D printed rectal swabs were compared with conventional flocked swabs and faecal samples, due to the potential advantages 3D printing as a technique offers for swab production and development. 16S rRNA gene sequencing, qPCR and metabolite profiling (using ^1^H-NMR spectroscopy) were performed on swab and faecal samples from healthy participants. Faecal calprotectin and total protein analysis were performed on samples from inflammatory bowel disease (IBD) patients. There were no significant differences between both swab types and faecal samples when assessing key measures of alpha and beta diversity, and differences in the abundance of major phyla. There was a strong correlation between both swab types and faecal samples for all combined metabolites detected by NMR. In IBD patients, there was no significant difference in faecal calprotectin and total protein levels between both swab types and faecal samples. These data lead us to conclude that 3D printed swabs are equivalent to flocked swabs for the analysis of the gut microbiome, metabolome and inflammation.

## Introduction

Investigation of the gut microbiome and metabolome, and of gastrointestinal diseases more generally, usually requires the provision of a faecal (or a mucosal biopsy) sample. However, there are often barriers to faecal sample collection for patients or trial participants including a sense that sample collection is unpleasant or unhygienic^1^. This barrier can result in a poor return rate for faecal tests, with rates as low as 35% being reported for faecal calprotectin tests in an inflammatory bowel disease (IBD) population^2^. Unlike other biosamples such as blood, faecal samples cannot always be easily provided when required, potentially introducing variability through transport and storage issues^3^. Obtaining mucosal biopsy samples generally requires a colonoscopy to be performed, with the attendant healthcare costs and risks, as well as the requirement for pre-procedure bowel cleansing, which is known to affect the gut microbiota^4^.

The use of rectal swabs is already well established for carbapenemase-producing Enterobacteriaceae (CPE) screening of at-risk patients in hospital settings^5^. Additionally, several studies have now shown good correlation between rectal swabs and matched faecal samples for more comprehensive profiling of gut microbiota composition using next generation sequencing^6–8^. There are also data which show that rectal swabs can be used to accurately profile key host and bacterial metabolites, again when compared to matched faecal samples^9^; this finding is of interest given the increased use of gut metabolome profiling as a key tool in investigating gut microbiome functionality^10^.

3D printing is a technique through which a physical object is produced from a 3-dimensional image using a 3D printer. It has previously been used in nasopharyngeal swab production, particularly during the Covid-19 pandemic, and is increasingly being evaluated for other potential healthcare applications^11,12^. It allows for rapid design modification and has the potential to produce swabs at a lower cost than existing flocked swabs. There are, however, no published studies on the use of 3D printed rectal swabs. In this study, 3D printed swabs (Instaswab, OPT Industries Inc.), flocked swabs (COPAN Floqswabs, COPAN) and matched faecal samples were compared for analysis of the gut microbiota and metabolome. Previous studies in this area have used similar flocked swabs^9^. The 3D swabs used consist of polymerised acrylic urethane polyethers within a computationally designed mesh, which optimises absorbance and elution (Supplementary Fig. 1). We also assessed the viability of both swab types for faecal calprotectin (FC) analysis (a commonly used marker of gastrointestinal inflammation), and for analysis of total protein levels^13^.

## Methods

### Study design and sample collection

This research and all experimental protocols were performed in accordance with institutional approval from the Research Governance and Integrity Team of Imperial College London, UK. Ethical approval was obtained from the Imperial College Research Ethics Committee (Reference: 21IC7228) and a UK Research Ethics Committee (ethical approval IRAS ID: 243310). Informed consent was obtained from all participants, and methods were carried out in accordance with relevant guidelines and regulations.

Five healthy controls (4 male, 1 female) were initially recruited. Exclusion criteria were any history of gastrointestinal disease or medications within the preceding three months. A crossover study design was followed with all participants providing four rectal swabs (two of each subtype) at two separate timepoints at least two weeks apart. Matched faecal samples were also provided at each timepoint in a Fecontainer™ (Excretas Medical BV, The Netherlands) and participants were asked to change the order the swabs were performed between the two collections. Detailed instructions on how to use both swab types and collect faecal samples were provided to all participants. All samples were collected on site at Imperial College London and transferred to storage at −80 ℃ within two hours of collection until analysis. This approach is consistent with suggestions from previous studies which have demonstrated alterations in the gut microbiome and metabolome in faecal samples kept at room temperature for prolonged periods of time^14,15^. In total 20 3D printed swabs, 20 flocked swabs and 10 faecal samples were collected. No formal sample size calculation was performed, but this number of swabs collected is consistent with similar previous studies^8,9^. A short questionnaire was given to all participants to briefly evaluate the acceptability of both swab types using a 5-point Likert scale (with responses ranging from strongly agree to strongly disagree; Supplementary Fig. 2). Participants were asked to use the scale to respond to statements regarding ease of use of each swab and whether either swab was uncomfortable.

For assessment of FC analysis and total protein extraction, 5 IBD patients (3 ulcerative colitis, 2 Crohn’s disease) had faecal samples collected for FC analysis. Swabs of both types were inoculated with faecal material by fully immersing the swab into the stool sample and stored at −80 ℃ until protein extraction and FC analysis.

### 16S rRNA gene sequencing and 16S rRNA gene qPCR analysis

DNA was extracted from swab and stool samples using the DNeasy PowerLyzer PowerSoil Kit (Qiagen, Hilden, Germany). Samples were homogenised in a Bullet Blender Storm instrument with a 10 min heating step to 65 ℃ step prior to beating. Sample libraries were prepared following Illumina’s 16S Metagenomic Sequencing Library Preparation Protocol using V1/V2 hypervariable region primers. V1/V2 primers were selected due to studies suggesting improved species identification compared to all other partial regions of the 16S rRNA gene, including V3/V4 sequencing^16,17^. Sequencing was performed using the Illumina MiSeq platform (Illumina Inc, Saffron Walden, UK) and the MiSeq Reagent Kit v3 (Illumina). Sequencing data was processed using the DADA2 pipeline (v1.18), as previously described, and the SILVA bacterial database^18^. Fastq files for the metataxonomic analysis have been deposited at the EBI’s ENA database under accession number PRJEB74914.

To quantify the bacterial biomass present, and to allow for analysis of the actual ecosystem abundance in each sample, 16S rRNA gene qPCR was performed using the DNA extracted from both swab types and faecal samples. For each reaction, a total of 20µL was made up, consisting of the following: 1 × KAPA2G Fast HotStart Ready Mix (Kapa Biosciences, Cat. No. KK5603) with ROX Reference Dye (20 µl/mL) (Biotium, Cat. No. 29052), 1.8 µM BactQUANT forward primer (5′ -CCTACGGGAGGCAGCA-3′), 1.8 µM BactQUANT reverse primer (5′ -GGACTACCGGGTATCTAATC-3′), 225 nM probe ((6FAM) 5′ -CAGCAGCCGCGGTA-3′ (MGBNFQ)), PCR grade water, and 5 µl DNA. Each plate included an *Escherichia coli* DNA (Sigma-Aldrich) standard curve in tenfold serial dilutions. All samples, standards, and controls were performed in triplicate. Extracted DNA samples were diluted to ensure they fell within the standard curve. The Applied Biosystems StepOnePlus Real-Time PCR System was used for amplification and real-time fluorescence detection.

Analysis of the 16S rRNA data was performed using MicrobiomeAnalyst (www.microbiomeanalyst.ca) Alpha diversity was assessed using Shannon’s diversity index, the Chao-1 index and Simpson’s index. Differences in alpha-diversity between groups was assessed using Kruskal–Wallis testing and p values were adjusted using the Benjamini–Hochberg method for false discovery rate (FDR). Differences in beta-diversity were assessed using the Bray–Curtis index after centre log-ratio data transformation by PERMANOVA, again with Benjamini–Hochberg FDR. A Kruskal–Wallis test with Dunn’s multiple comparisons test was used to compare 16S rRNA gene copy number between sample groups using GraphPad Prism (v9.5.1). For analysis of differences at the level of the phyla and genus a Kruskal–Wallis test was performed with Benjamini–Hochberg FDR in RStudio (Version 2023.12.1 + 402).

### ^1^H-Nuclear magnetic resonance spectroscopy (NMR) analysis

Faecal samples were thawed and homogenised before a faecal water was made by adding ultra high-performance liquid chromatography water (UHPLC) in a 2:1 ratio. Samples were vortexed for 5 min before being centrifuged at 20,000 × *g* at 4 ℃ for 20 min and the supernatant transferred into a new microcentrifuge tube with 1.5 M KH_2_PO_4_ buffer (pH 7.4, 100% of deuterium oxide (D_2_O), 2 mM sodium azide, and 1% of TSP [3-trimethylsilyl-[2,2,3,3,-^2^H_4_]-propionic acid sodium salt]) in a 9:1 ratio. This mixture was briefly vortexed and centrifuged, before being transferred into 3 mm NMR tubes.

For the rectal swabs, the swab head was broken off at the pre-marked break point (2 cm from the swab tip) and placed into a microcentrifuge tube. An aliquot (400 µL) of 1.5 M KH_2_PO_4_ buffer-UHPLC H_2_O mixture (in a 1:9 ratio) was added to the tube, and the samples were vortexed for 30 s and sonicated for 30 s. These samples were centrifuged at 20,000 × *g* at 4 ℃ for 20 min. The swab tip was removed, samples were briefly centrifuged, and the supernatant was transferred into 3 mm NMR tubes. 20 µL of each sample (from both swabs and stool samples) was pooled to create a quality control (QC) sample for calibration.

^1^H-NMR spectroscopy was performed using the Bruker AVANCE III HD 600 MHz spectrometer (Bruker Bio-Spin, Rheinstetten, Germany) using a 5 mm CPTCI 1H-13C/15 N/D Z-gradient cryoprobe. 1D spectra (a standard NOESYGPPR1D pulse sequence (RD-90°-t1-90°-tm-90 -ACQ)) as well as 2D spectra (standard 2D JRESGPPRQF pulse sequence) were acquired. A recycle delay of 4 s and mixing time of 100 ms was used. The 90° pulse length was ~ 10 μs.

Analyses of NMR data were performed using GraphPad Prism (v9.5.1) following probabilistic quotient normalisation (PQN). Metabolite annotation was performed using statistical total correlation spectroscopy (STOCSY) in Chenomx Software (Alberta, Canada)^19^. Pearson’s correlation was used to compare all metabolite values between sample groups and individual metabolite differences were assessed using a one-way ANOVA with Tukey’s test for multiple comparisons.

### Faecal Calprotectin analysis

Calprotectin concentration was determined using the Accusay Calprotectin Plus™ kit (Launch Diagnostics, UK) per manufacturer’s instructions. Both swabs and 90 mg of matched faecal sample were transferred to sterile 15 ml tubes with 4.4 ml extraction buffer and mechanically homogenised for 30 min on a multi-tube vortex. The handles of the swabs and inoculation loops inside the tubes acted as an agitator. An aliquot (1 ml) of suspension was transferred to sterile microcentrifuge tubes and centrifuged for 20 min at 10,000 × *g*. Supernatant (800 μl) was extracted and diluted to 1:400. Diluents (100 μl) were pipetted in triplicate on an antibody-coated 96 well ELISA plate along with six calibrators with known calprotectin concentrations ranging from 0 to 2800 μg/g and controls (high and low). The plate was covered and incubated for 60 min at 20 ℃ and washed three times. Enzyme Conjugated Antibody (100 μl) (Horseradish peroxidase-labelled mouse anti-human calprotectin IgG antibodies in a buffer solution with Proclin-300 preservative) was added to each well and further incubated for 30 min at 20 °C and washed three times again. Substrate TMB (100 μl)(3,3’,5,5’-Tetramethylbenzidine) was added to each well and incubated for 15 min in the dark due to TMB photosensitivity. Finally, stop solution (100 μl) containing H2SO4 (0.5 M) was added. The oxidised TMB was measured at 450 nm using MultiSkan™Go Microplate Spectrophotometer (ThermoFisher Scientific, UK) and processed on SkanIT Software (v7.0.2).

Comparison of results between sample types was performed using a Kruskal–Wallis test followed by Dunn’s test for multiple comparisons, using GraphPad Prism (v9.5.1).

### Total protein analysis

Protein concentration was determined using the Pierce™ BCA (Bicinchoninic acid assay) Protein Assay Kit (ThermoFisher Scientific, UK) per manufacturer’s instructions. Both swabs and 250 mg matched faecal samples were eluded in 1 ml phosphate buffered saline (PBS) and mechanically homogenised for 5 min. The homogenate was transferred to sterile microcentrifuge tubes and centrifuged for 20 min at 20,000 × *g*. 100 μl of a tenfold and 100-fold dilution of each supernatant sample was made in sterile PBS. Standards were prepared by diluting a stock solution with known concentration of bovine serum albumin to a series of 9 concentrations ranging from 0 to 2000 μg/μl. An aliquot of the standards and samples (25 μl, undiluted, tenfold and 100-fold dilution) were pipetted in triplicate into sterile Nunclon™ DeltaSurface 96well plates (ThermoFisher Scientific, Denmark). The BCA working reagent was prepared according to manufacturer instructions and 200 μl was pipetted into all wells of the 96 well plate. The plates were incubated for 30 min at 37 ℃ and allowed to cool to room temperature. Colorimetric change was measured at 562 nm using MultiSkan™Go Microplate Spectrophotometer (ThermoFisher Scientific, UK) and results processed on SkanIT Software (v7.0.2).

Comparison of results between sample types was performed using a Kruskal–Wallis test followed by Dunn’s test for multiple comparisons on GraphPad Prism (v9.5.1).

## Results

### Microbiome diversity and composition show no significant differences between both swab types and faecal samples

**Figure 1:**
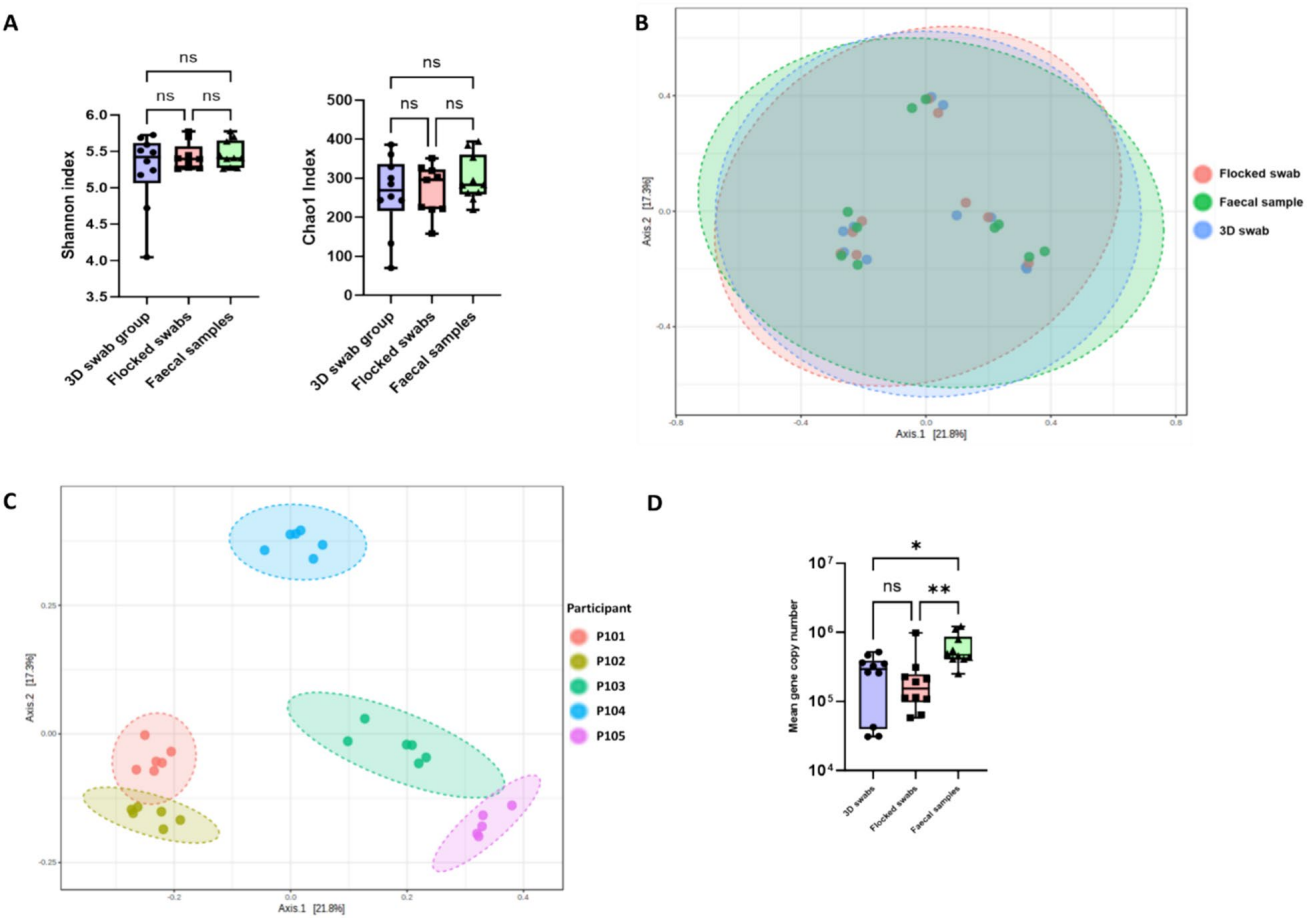
(**A**)—Alpha diversity using Shannon and Chao1 index by sample type (p-values: 0.750, 0.762; [Kruskal–Wallis]), (**B**)—Beta diversity by sample type (PERMANOVA: F-value: 0.595; R-squared: 0.0437; p-value: 0.951), (**C**)—Beta-diversity by participant (PERMANOVA: F-value: 9.00; R-squared: 0.600; p-value: 0.001 ), (**D**)—16S rRNA gene qPCR data compared between sample types.

There was no significant difference between sample types when assessing alpha diversity using Shannon’s diversity index (p = 0.750) (Fig. 1A), the Chao1 index (p = 0.762) or Simpson’s index (p = 0.832). Beta-diversity showed differences between individuals, but not between sample subtype i.e. stool vs 3D swab vs flocked swab. When assessing beta diversity between sample type using the Bray–Curtis index, a PERMANOVA analysis showed no significant difference (p = 0.951) (Fig. 1B). Between individuals, again using PERMANOVA, there was a significant difference (p = 0.001) (Fig. 1C). Analysis of 8 major bacterial phyla (including Bacteriodota, Bacillota and Pseudomonadota) showed no significant differences between the sample groups. Additionally, at the genus level there was no statistically significant difference for 78/84 genera analysed. When applying a low count filter requiring a minimum count of 5 and a 10% prevalence across all samples, 3D swabs identified 78 bacterial genera, flocked swabs 81 and faecal samples 76. Analysis of bacterial biomass using qPCR analysis showed no significant difference between the two swab subtypes (p > 0.999), but a significant difference between both swab types and faecal samples (faecal vs 3D swabs p = 0.0384, faecal vs flocked swabs p = 0.0038) albeit less so with the 3D printed swabs than flocked swabs (Fig. 1D).

### NMR data show good correlation in metabolite levels between both swab types

**Figure 2:**
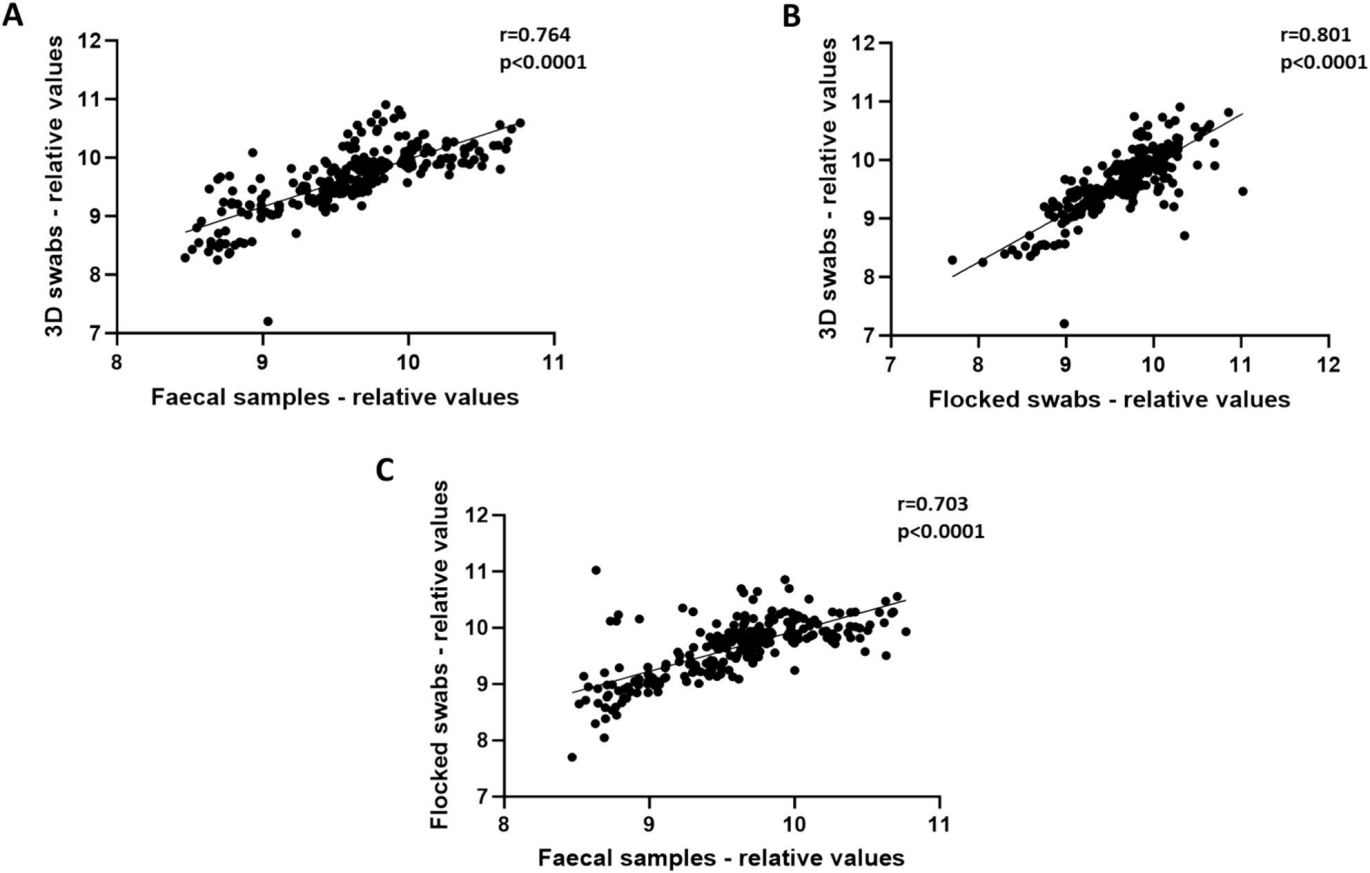
Correlation of all relative abundance values for analysed metabolites (Pearson’s correlation coefficient on PQN normalised and log transformed data). (**A**) – 3D swabs vs faecal samples. (**B**) – 3D swabs vs flocked swabs. (**C**) – faecal samples vs flocked swabs.

A total of 27 individual metabolites were identified for analysis (Supplementary Table 1). Correlation analysis of all metabolite levels using Pearson’s correlation coefficient showed a strong correlation between 3D swabs and faecal samples (r = 0.764) and between 3D swabs and flocked swabs (r = 0.801) **(**Fig. 2A, 2B**)**. Flocked swabs also correlated strongly with faecal samples (r = 0.703) (Fig. 2C). A one-way ANOVA with Tukey’s multiple comparison test showed no significant differences for 25/27 individual metabolites when comparing the two swab types. When comparing to faecal samples, both swabs showed no significant differences for most metabolites (22/27 for flocked swabs, 16/27 for 3D swabs) (Supplementary Table 1).

### Both swab types show good correlation with faecal samples for measuring faecal calprotectin and total protein levels

**Figure 3:**
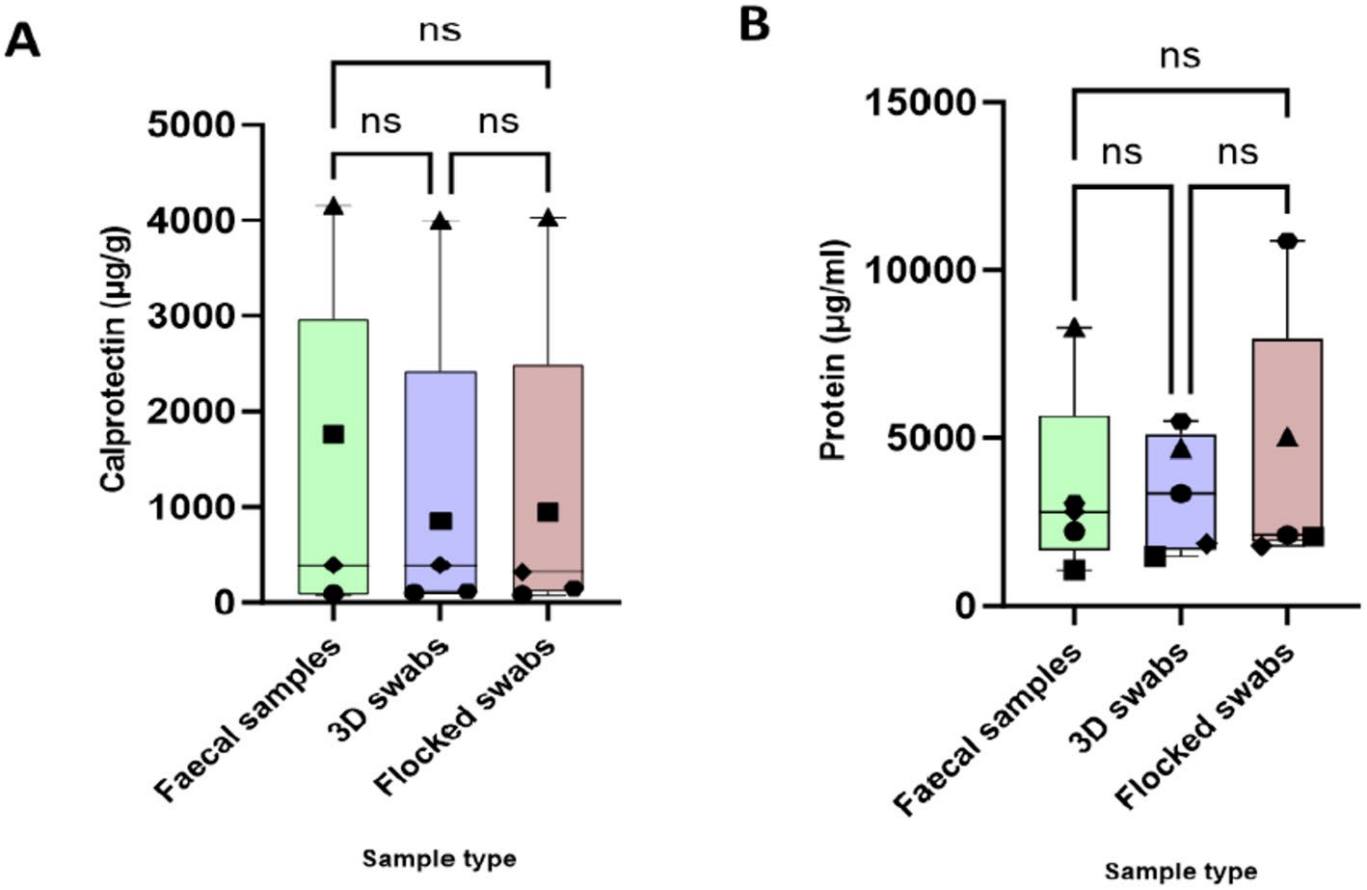
(**A**) – Faecal Calprotectin test results by sample type. (**B**)—Total protein concentration from BCA assay by sample type.

All calprotectin tests were positive when using a cut-off of 50 µg/g as stipulated by the National Institute for Clinical Excellence guidelines (mean 1165.7 µg/g, range 79.8-4158 µg/g), consistent with the diagnosis of active IBD in all recruited patients^20^. There were no significant differences between calprotectin levels across the three sample subtypes (p = 0.95) (Fig. 3A). In addition, when using a cut-off of 50 µg/g, none of the numerical differences between sample types in the same patient would have crossed this diagnostic threshold: the slight differences would be considered clinically insignificant. The results from the BCA assay (looking at total protein extracted) show a similar picture – again, there was no statistically significant difference between groups (p > 0.99) (Fig. 3B).

### A majority of participants felt both swab types were acceptable to use

When scored for comfort on the Likert scale, swab types were equivalent with only 1/5 participants feeling that both the 3D printed and flocked swabs were “uncomfortable to use”. Most participants agreed that both swabs were easy to use with only 1/5 participants disagreeing with the statement “the 3D printed swab was easy to use”, providing a free text comment that they felt the swab was too short. Other free text comments provided included suggestions that the 3D printed swab could be made softer and the break point on the swab was too high, making it easy to go past.

## Discussion

This study aimed to compare the performance of 3D printed rectal swabs to existing flocked swabs and faecal samples for analysis of the gut microbiome, metabolome, FC measurement and total protein measurement. 3D printed swabs seem equivalent to the existing flocked swabs currently used in healthcare settings for analysis of the gut microbiome (and, in research settings, the metabolome). Both swab types correlated well with the results from matched faecal samples for 16S rRNA gene sequencing analysis of the gut microbiome across several measures. This finding is consistent with previous data demonstrating that rectal swabs correlate with faecal samples in various settings including healthy participants, patients with cirrhosis and patients in intensive care units^6–8,21,22^. NMR results showing a strong correlation between both swab types and stool are again consistent with previous studies (9). Some differences could be seen between both swab types and faecal samples when analysing individual metabolites, as again has been described previously, but significant differences were minimal between the two swab subtypes^9^.

Analysis of both swab types’ potential for use in the collection of faecal calprotectin (FC) samples showed good correlation between results from both swab types and faecal samples. Measurement of FC and total protein involved inoculation of both swab types in stool samples provided by IBD patients and so further data is therefore needed in real-world settings, following a variety of different collection protocols, to validate whether rectal swabs have a role in clinical practice for this purpose. However, with previous studies showing poor compliance with FC testing in IBD patients, investigation of alternative methods of FC collection is of interest^2^. The comparable BCA assay results between swab and faecal samples suggest that the measurement of other clinically relevant proteins may be possible if adequate collection protocols can be developed.

Overall, these data add to the evidence supporting the potential use of rectal swabs as an alternative method for investigation and diagnosis of gastrointestinal disease and a role for swabs produced by 3D printing. Our finding that 3D printed swabs are equivalent to flocked swabs is important, as 3D printing allows for rapid design modification, meaning that 3D swab design can easily be optimised to allow for improved sampling in future. Design flexibilitywould not be readily possible with conventionally manufactured flocked swabs. In addition, whilst many factors influence swab production cost, previous studies have suggested that 3D printing can be a cost-effective method of swab production^23^. Further research, investigating how physical modifications and material changes in 3D printed swabs can influence their performance, would also be of interest. Our survey of swab acceptability leads us to suggest that both swab types were acceptable to participants. Some minor suggestions were made regarding the design of the 3D swabs, however as discussed, the ease of design modification in 3D printing would make it straightforward to make these changes in future swab production.

The main limitation of our study is the relatively small sample size; a longitudinal sampling study design has been used to compensate for this issue. However, further research is still required in larger cohorts to fully validate the use of 3D printed rectal swabs, and to establish the exact role for rectal swab use more generally in both clinical and research settings.

To conclude, these results show that 3D printed swabs are equivalent to existing flocked swabs. Our data confirm that rectal swabs more generally may have a role as an alternative method of sample collection to analyse the gut microbiota. We also conclude that there may be a role for the use of rectal swabs in the measurement of faecal calprotectin and other significant proteins in gastrointestinal disease.

### Supplementary Information

Supplementary Information.

## Data Availability

Fastq files for the metataxonomic analysis have been deposited at the EBI’s ENA database under accession number PRJEB74914.
