# Supplementary material for: 3D printed rectal swabs for assessing the gut microbiome, metabolome and inflammation
Source: Sci Rep. 2024 Jul 18;14:16613. doi: 10.1038/s41598-024-67457-0 (PMC11258137; doi:10.1038/s41598-024-67457-0)
Supplement: Supplementary file 1 — Supplementary Information. [file 41598_2024_67457_MOESM1_ESM.docx]

**3D Printed Rectal Swabs for Assessing the Gut Microbiome, Metabolome and Inflammation – Supplementary Data**

**Supplementary data:**


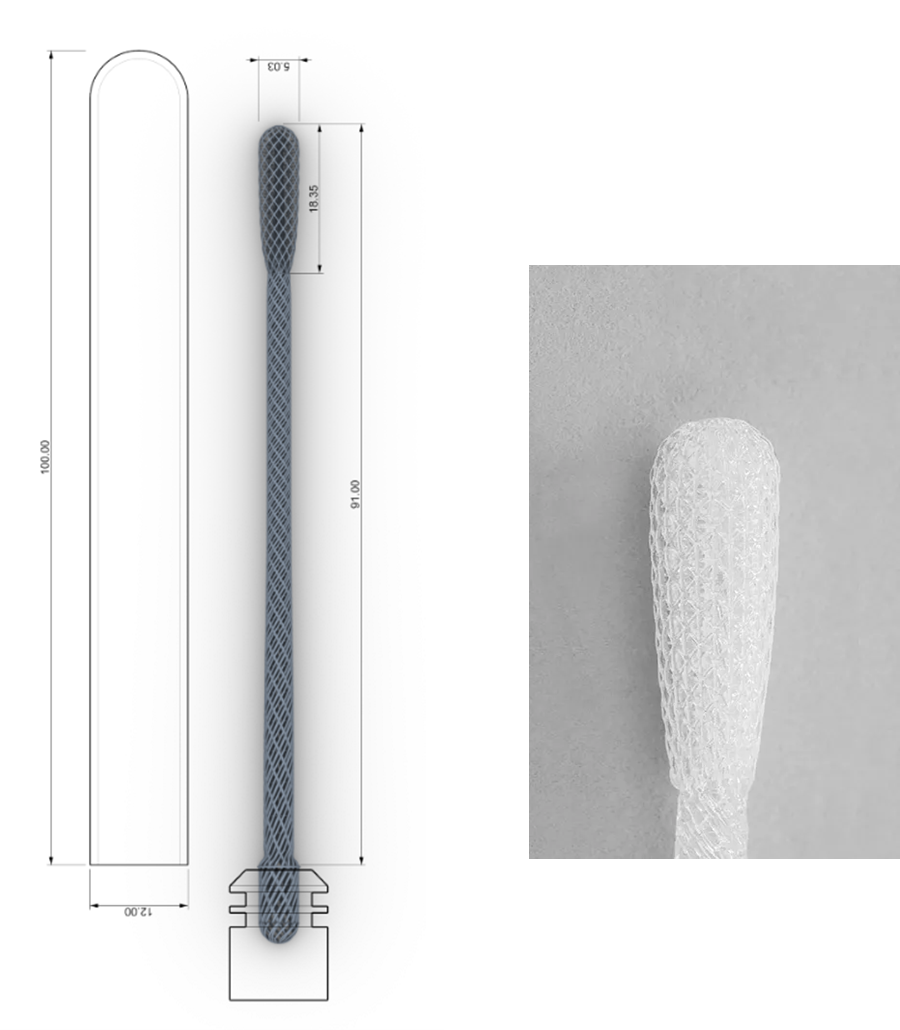


**Supplementary Figure 1:** Dimensions and image of 3D printed rectal swab used. Measurements in millimetres.


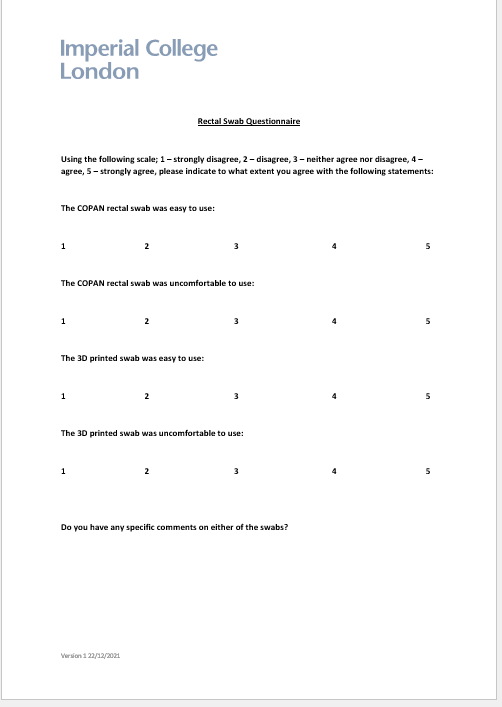


**Supplementary Figure 2:** Questionnaire used to assess acceptability of both swab types to participants.


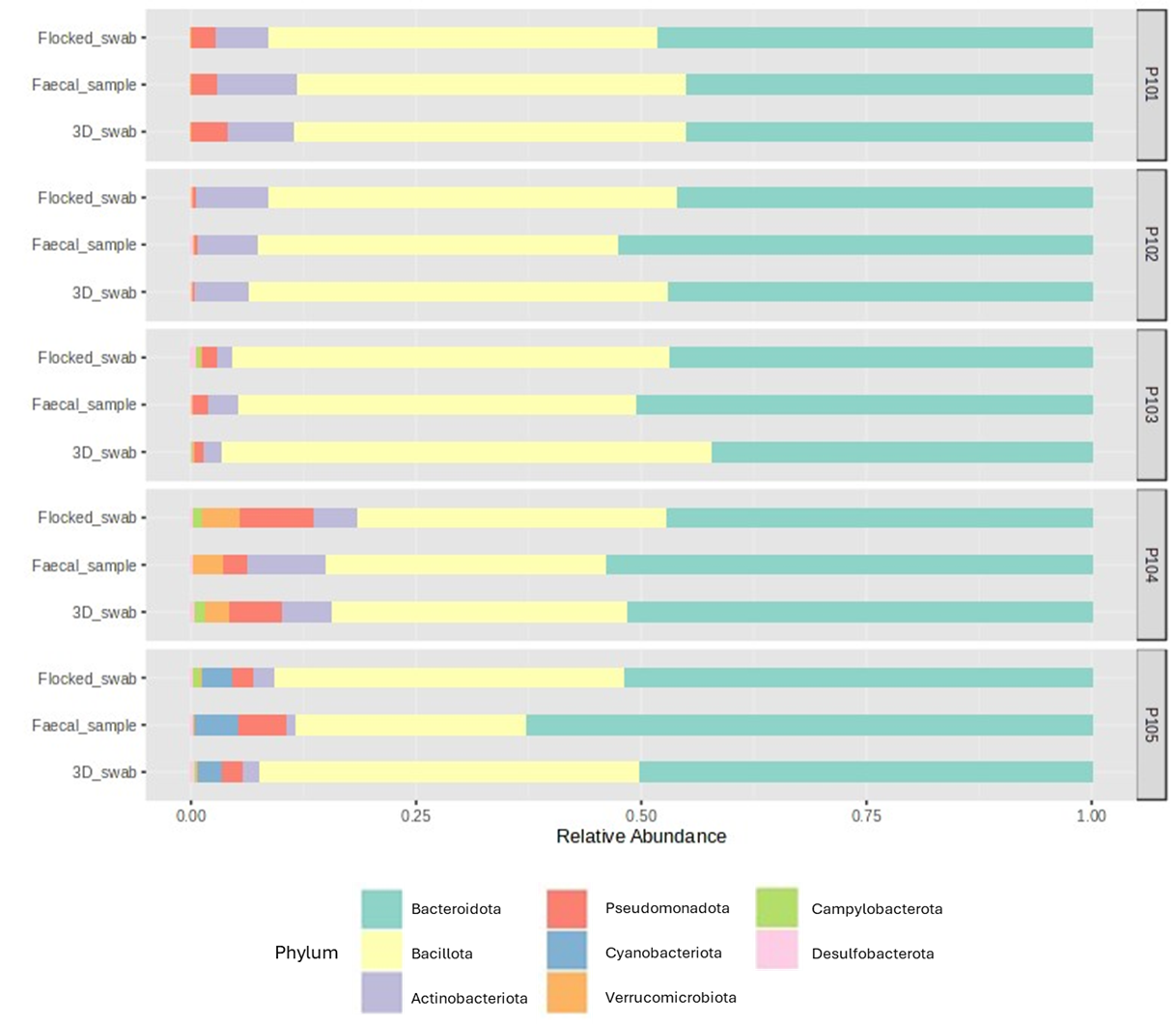


**Supplementary figure 3**: Relative abundance by phylum. Grouped by participant and sample type.


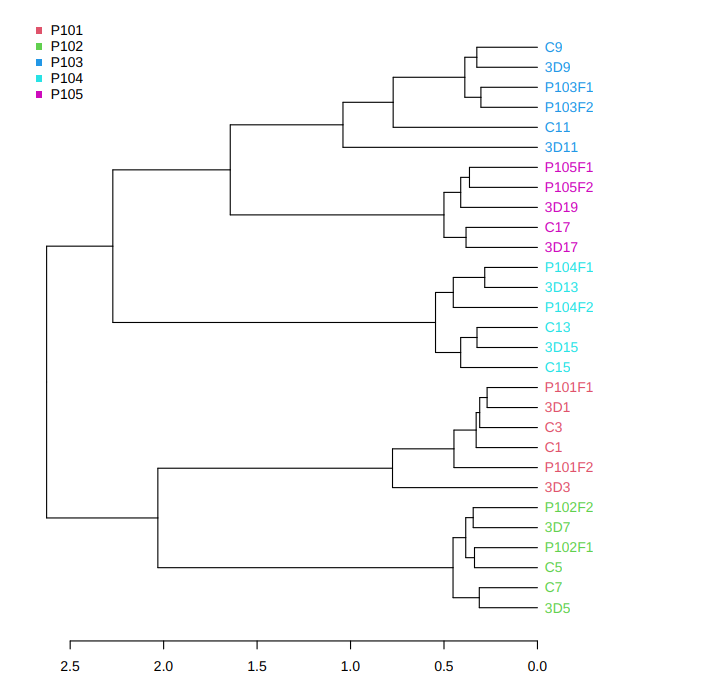


**Supplementary figure 4**: Beta-diversity dendrogram by participant using Bray-Curtis index

**Supplementary table 1**: Results of one-way ANOVA for both swab types compared to faecal samples and to each other for individual metabolites. ****p<0.0001, ***p<0.001, **p<0.01, *p<0.05, ns = non-significant

| **Metabolite** | **Flocked swabs vs Faecal samples** | **3D swabs vs Faecal samples** | **Flocked swabs vs 3D swabs** |
| --- | --- | --- | --- |
| Acetate | ** | ** | ns |
| Acetoin | ns | ns | ns |
| Alanine | ns | * | ns |
| Arginine | ns | ns | ns |
| Aspartate | ns | ns | ns |
| Butyrate | * | * | ns |
| Formate | ns | ns | ns |
| Fucose | ** | ns | ns |
| Fumarate | ns | ns | ns |
| Glucose | ns | ns | ns |
| Glutamate | ns | ns | ns |
| Glycine | ns | ns | ns |
| Hypoxanthine | ns | * | ns |
| Isoleucine | ns | ** | ns |
| Lactate | ns | ns | ns |
| Leucine | ns | ns | * |
| Methionine | ns | ns | ns |
| Nicotinate | ns | ns | ns |
| Phenylalanine | ns | * | ns |
| Propionate | ** | ** | ns |
| Pyruvate | * | ns | ns |
| Succinate | ns | ns | ns |
| TMA | ns | * | ns |
| Tryptophan | ns | * | ns |
| Tyrosine | ns | ns | ns |
| Uracil | ns | * | * |
| Valine | ns | * | ns |
